# Comparison of balanced crystalloids versus normal saline in patients with diabetic ketoacidosis: a meta-analysis of randomized controlled trials

**DOI:** 10.3389/fendo.2024.1367916

**Published:** 2024-05-21

**Authors:** Yuting Liu, Jianfeng Zhang, Xiaoya Xu, Xiaoyun Zou

**Affiliations:** ^1^ Oncology and Chemotherapy Department, The First Affiliated Hospital of Lishui University, Lishui People’s Hospital, Lishui, China; ^2^ Department of Orthopedics, Yunhe People’s Hospital, Yunhe, China; ^3^ Department of General Surgery, The First Affiliated Hospital of Lishui University, Lishui People's Hospital, Lishui, China; ^4^ Department of General Practice, The First Affiliated Hospital of Lishui University, Lishui People’s Hospital, Lishui, China

**Keywords:** balanced crystalloids, normal saline, diabetic ketoacidosis, meta-analysis, resuscitation

## Abstract

**Purpose:**

The optimal resuscitative fluid for patients with diabetic ketoacidosis (DKA) remains controversial. Therefore, our objective was to assess the effect of balanced crystalloids in contrast to normal saline on clinical outcomes among patients with DKA.

**Methods:**

We searched electronic databases for randomized controlled trials comparing balanced crystalloids versus normal saline in patients with DKA, the search period was from inception through October 20^th^, 2023. The outcomes were the time to resolution of DKA, major adverse kidney events, post-resuscitation chloride, and incidence of hypokalemia.

**Results:**

Our meta-analysis encompassed 11 trials, incorporating a total of 753 patients with DKA. There was no significant difference between balanced crystalloids and normal saline group for the time to resolution of DKA (MD -1.49, 95%CI -4.29 to 1.31, P=0.30, I^2^ = 65%), major adverse kidney events (RR 0.88, 95%CI 0.58 to 1.34, P=0.56, I^2^ = 0%), and incidence of hypokalemia (RR 0.80, 95%CI 0.43 to 1.46, P=0.46, I^2^ = 56%). However, there was a significant reduction in the post-resuscitation chloride (MD -3.16, 95%CI -5.82 to -0.49, P=0.02, I^2^ = 73%) among patients received balanced crystalloids.

**Conclusion:**

Among patients with DKA, the use of balanced crystalloids as compared to normal saline has no effect on the time to resolution of DKA, major adverse kidney events, and incidence of hypokalemia. However, the use of balanced crystalloids could reduce the post-resuscitation chloride.

**Systematic review registration:**

https://osf.io, identifier c8f3d.

## Introduction

Diabetic ketoacidosis (DKA) is a life-threatening complication of diabetes mellitus described in patients with both type 1 and type 2 diabetes (1). The pivotal facets of acute DKA management involve the administration of intravenous fluids and insulin therapy (2, 3). Despite a consensus on the appropriate insulin dosage and administration route, the selection of fluid therapy remains a subject of contention, particularly in the context of both DKA and critical illness (4, 5).

For decades, the normal saline (0.9% sodium chloride solution) has been the most commonly administered crystalloid solution worldwide (5, 6). The current international guidelines also recommend normal saline as the replacement fluid of choice for DKA (7, 8). However, recent findings raise concerns regarding the potential consequences of normal saline administration, including heightened acidemia, diminished renal blood flow, and reduced urine output (9). Such alterations may culminate in acute kidney injury (AKI) (10–12) and mortality (13, 14). Since the high chloride content of normal saline may lead to possible adverse effects, balanced crystalloids, in which chloride anions are replaced by lactate or acetate, are increasingly used alternatives (15). These alternatives, wherein chloride anions are substituted with lactate or acetate, present a chemical composition more akin to human plasma than normal saline. This compositional alignment involves reduced chloride levels and an augmented *in vivo* strong ion difference (16).

Currently, the preferred choice between normal saline and balanced crystalloids in patients with DKA remains a subject of debate. In view of the widespread use of intravenous fluids therapy worldwide, even minor distinctions in fluid selection and their implications for clinical outcomes carry substantial clinical significance. Consequently, the aim of this meta-analysis was to scrutinize the repercussions of utilizing balanced solutions in contrast to 0.9% saline solutions on clinical outcomes in patients with DKA.

## Methods

We performed our meta-analysis following the guidelines of updated PRISMA statement (17) (**Supplementary Material 1**). The protocol of this meta-analysis has been registered in the Open Science Framework (https://osf.io/c8f3d). A systematic exploration for eligible randomized controlled trials (RCTs) in the English language was conducted through an extensive literature search across four electronic databases (PubMed, Embase, Scopus, and Cochrane Library), spanning from their inception through October 20th, 2023. The literature search was conducted through the utilization of keywords comprising “balanced crystalloids”, “normal saline”, “DKA”, and “randomized”. The comprehensive search methodologies are detailed in **Supplementary Material 2**.

### Eligibility criteria

The inclusion criteria were shown as follows: 1. Design: randomized trials; 2. Population: patients with DKA, the definition of DKA was according to the current international guidelines (7, 8); 3. Intervention: balanced crystalloids characterized by a chloride concentration closely approximating physiological levels (e.g., Lactated Ringer’s, Plasma-Lyte); 4. Comparison: normal saline, specified as 0.9% saline with a chloride content of 154 mmol/L; 5. Outcomes: primary outcome were the time to resolution of DKA, defined by the original authors, major adverse kidney events (defined as KDIGO stage II or higher (18), or receipt of renal-replacement therapy, or defined by the original authors), post-resuscitation chloride, and incidence of hypokalemia (potassium < 3.0 mmol/L).

### Data extraction and quality assessment

Two authors (Yuting Liu, Jianfeng Zhang) conducted the retrieval of relevant studies. Reports considered potential for inclusion were screened in full text. Differences in this process were resolved by consensus. When no consensus was reached, a third co-author (Xiaoya Xu) would resolve the issue. Standardized form from the Cochrane Data Collection template was adapted and used to create a study-specific data abstraction form, data including the first author, publication years, study design, sample size, population characteristics, and details pertaining to the intervention and control agents were independently extracted by two authors (Yuting Liu, Jianfeng Zhang). Predefined outcomes from the included studies were also extracted. If data were not available in the trial report or data collection, we contacted the corresponding authors to provide important missing data. In instances where studies reported continuous outcomes in the form of median and interquartile range, we used the median, interquartile range, and sample size to estimate the approximate mean value and standard deviation. The calculating formula were proposed by Wan et al. (19), they also developed a software to estimate the mean value and standard deviation.

The Cochrane risk of bias tool (20) was used for assessing the methodological quality by two authors (Yuting Liu, Jianfeng Zhang). Discrepancies in assessments were resolved through consultation with a third co-author (Xiaoya Xu).

### Statistical synthesis and analysis

The risk ratios (RRs) with corresponding 95% confidence intervals (CI) were calculated by using a Mantel and Haenszel model for dichotomous outcomes. The mean difference (MD) with 95% CI were calculated by using an inverse variance model for continuous outcomes. Pooled estimates are displayed in forest plots. The evaluation of heterogeneity among studies relied on Higgins inconsistency (I^2^) statistics (21), with substantial heterogeneity identified when the I^2^ value exceeded 50%. In the absence of significant heterogeneity, a fixed-effects model was employed for analysis; otherwise, a random-effects model was applied. Furthermore, an assessment of publication bias was conducted using the funnel plot. Additionally, a sensitivity analysis was executed to investigate the impact of individual studies through the successive exclusion of each study.

In order to control the type-I and II errors, we performed *post hoc* trial sequential analyses (TSA) by using the TSA software (0.9.5.10 Beta, The Copenhagen Trial Unit, Denmark). The parameters used in TSA were detailed in **Supplementary Material 2**.

## Results

### Study identification and characteristics

**Figure 1:**
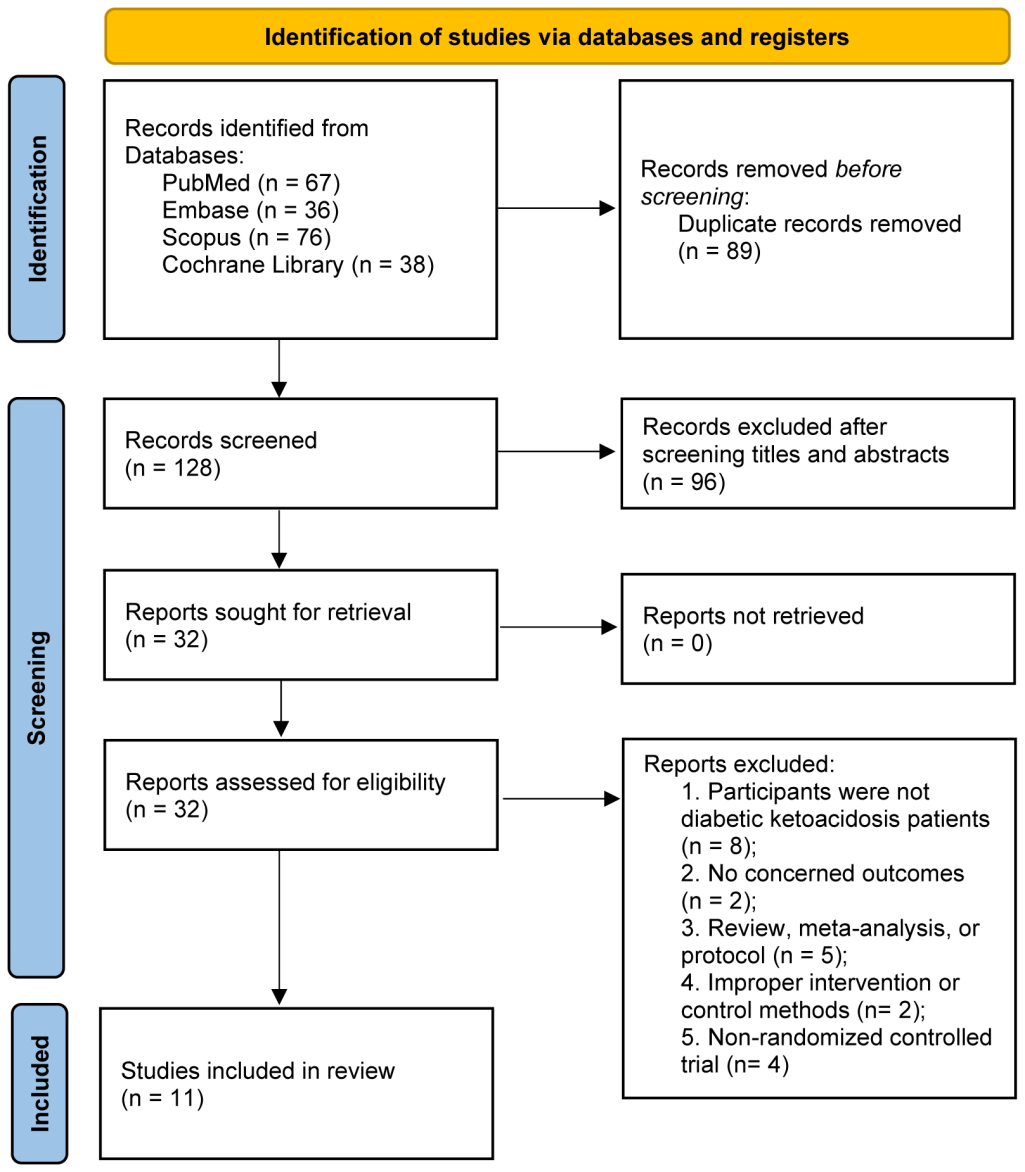
PRISMA 2020 flow diagram for the meta analysis.

In the initial phase of the search process, 217 articles were identified. First, all records were imported into a document management software, and 89 duplicated literatures were electronically removed. After reading the titles and abstracts, 96 studies were further excluded. During the evaluation of the full text, 21 studies were excluded for specific reasons (**Supplementary Material 2** recorded the list of excluded studies with reasons). Finally, our study included a total of 11 RCTs (22–32), (flow chart presented in **Figure 1**).

**Table 1** delineates the attributes of the studies incorporated in our analysis. The analysis encompassed a total of 753 patients, with 392 patients subjected to balanced crystalloids and 361 patients administered normal saline throughout the study duration. The patient count across individual studies exhibited variability, ranging from a minimum of 30 to a maximum of 172. Notably, the studies included in our analysis exhibited diversity in terms of study populations: nine trials (22–25, 27, 28, 30–32) included adult patients with DKA, and two trial (26, 29) included patients in medical ICU. Different intervention drugs were also identified: Plasma-Lyte in six trials (22, 27–30, 32), Lactated Ringer’s solution in four trials (23–25, 32), and Hartmann’s solution in one trial (26). Apart from trial fluid administered, there were no other changes to the standard DKA treatments including insulin, electrolyte replacement, and/or supportive management.

**Table 1:** Characteristics of included studies.

Study	Design	No. of patients	Population	Intervention	Outcomes
Mahler 2011 (22)	Double-blind, single-center	22/23	Patients aged 18 to 65 years with moderate to severe DKA in the ED	Plasma-Lyte A versus 0.9% NS, each patient received a 20 mL/kg bolus of study fluid. The remaining volume deficit was replaced over 24 hours	Post-resuscitation chloride
Van Zyl 2011 (23)	Double-blind, multicenter	27/27	Patients aged more than 18 years with DKA	Lactated Ringer’s solution versus 0.9% NS, until the blood glucose was < 14 mmol/L	Time to DKA resolution (pH > 7.3, serum bicarbonate ≥ 18 mmol/L, blood glucose < 11.1 mmol/L)
Semler 2017 (25)	Open-label, multicenter	21/20	All adults with severe DKA admitted to the ICU	Lactated Ringer’s solution or Plasma-Lyte versus 0.9% NS, until discharge from the ICU	Major adverse kidney event
Yung 2017 (26)	Double-blind, multicenter	38/39	Children with moderate to severe DKA admitted to the PICU or high-dependency unit	Hartmann’s solution versus 0.9% NS in bolus of 10 to 30 mL/kg for at least 12 hours	Time to DKA resolution (pH > 7.3, serum bicarbonate ≥ 15 mmol/L)
Aditianingsih 2017 (24)	Single-blind, single-center	15/15	Patients aged 18 to 65 years with DKA in the ED	Lactated Ringer’s solution versus 0.9% NS, until the resolution of DKA	Major adverse kidney event
Tsui 2019 (27)	Open-label, single-center	22/20	Patients aged more than 18 years with DKA in the ED	Plasma-Lyte A versus 0.9% NS, until the resolution of DKA	Time to DKA resolution (pH > 7.3, serum bicarbonate ≥ 15 mEq/L, blood glucose < 200 mg/dL, anion gap < 12)
Self 2020 (28)	Double-blind, multicenter	94/78	Patients aged 18 years or older with DKA in the ED and ICU	Ringer lactate solution or Plasma-Lyte A versus 0.9% NS, until discharge from the ED or ICU	Time to DKA resolution (pH > 7.3, serum bicarbonate ≥ 15 mEq/L, blood glucose < 200 mg/dL), major adverse kidney event, hypokalemia
Williams 2020 (29)	Double-blind, single-center	34/32	Children aged from 1 month to 12 years with DKA in the pediatric emergency room	Plasma-Lyte A versus 0.9% NS, until the resolution of DKA	Time to DKA resolution (pH > 7.3, serum bicarbonate > 15 mEq/L, blood glucose < 200 mg/dL), major adverse kidney event, post-resuscitation chloride, hypokalemia
Ramanan 2021 (30)	Open-label, multicenter	48/42	Adult patients aged more than 16 years with severe DKA in the ED or ICU	Plasmalyte-148 versus 0.9% NS for 48 hours	Post-resuscitation chloride, major adverse kidney event, hypokalemia
Yan 2023 (32)	Triple-blind, single-center	25/27	Adult patients aged more than 18 years with DKA in the ED	Ringer lactate solution versus 0.9% NS, until the resolution of DKA	Time to DKA resolution (pH > 7.3, serum bicarbonate ≥ 15 mEq/L, blood glucose < 200 mg/dL, anion gap < 12), major adverse kidney event, hypokalemia
Attokaran 2023 (31)	Open-label, multicenter	46/38	Adult patients with DKA in the ED	Plasmalyte-148 versus 0.9% NS, until discharge from the ED	Post-resuscitation chloride

DKA, diabetic ketoacidosis; ED, emergency department; NS, normal saline; ICU, intensive care unit; PICU, pediatric intensive care unit.

### Quality assessment

**Figure 2:**
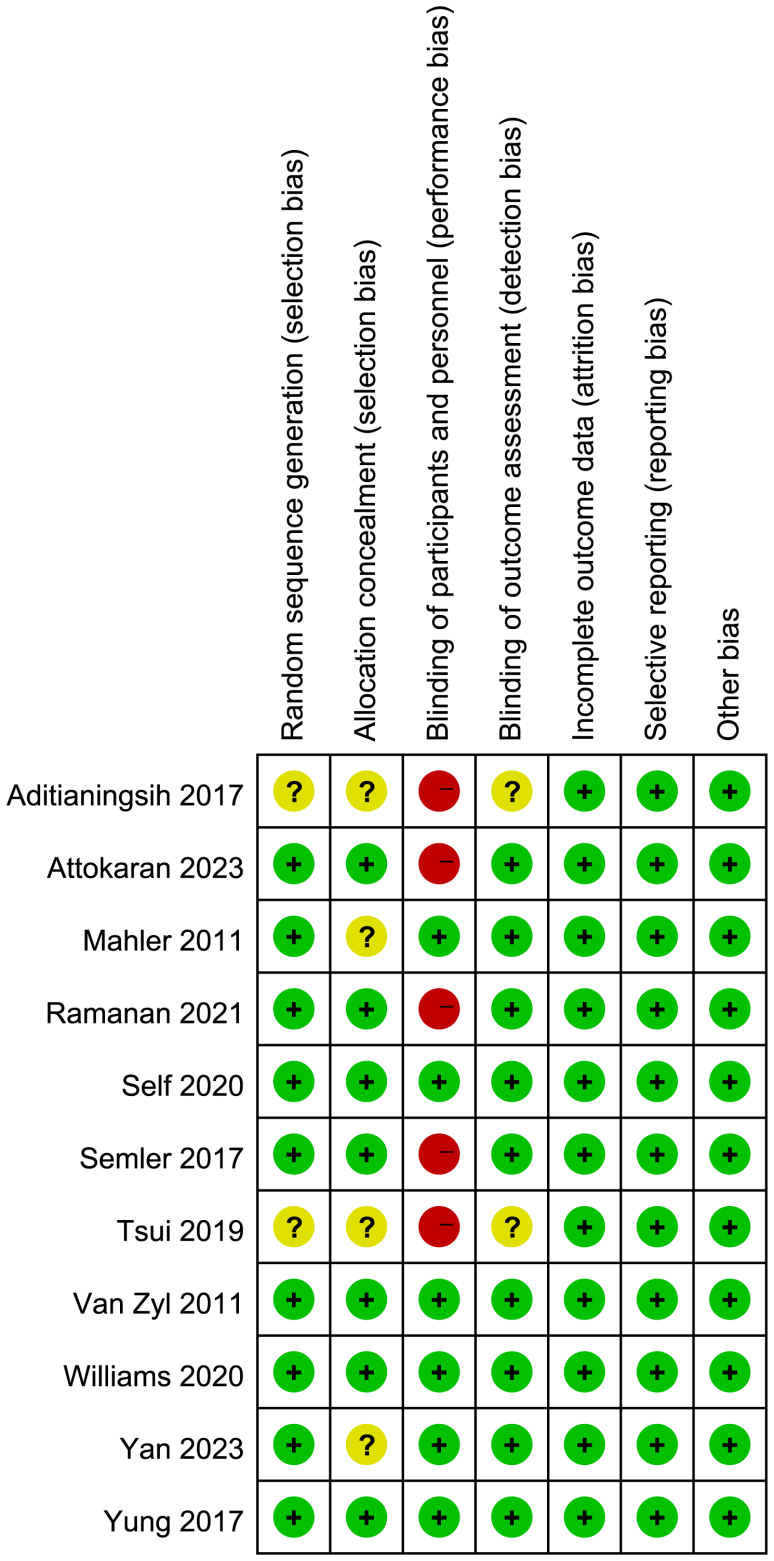
Assesment of quality by the Cochrane risk of bias tool. Red denotes high risk, yellow unclear risk and green low rsik.

**Figure 2** presents the quality assessment by the Cochrane risk of bias tool. Notably, five trials (24, 25, 27, 30, 31) were single-blind or open-label trials, they had high risk of bias. Additionally, four studies (22, 24, 27, 32) did not report the details of random sequence generation or allocation concealment. Regarding the blinding method for outcome assessment, two trials (24, 27) exhibited an unclear risk of bias, introducing potential variability in the size of the observed effect. The detailed description of quality assessment was reported in **Supplementary Material 2**. The evaluation of publication bias, as illustrated by the funnel plot (**Supplementary Material 2**), did not indicate a significant risk of publication bias.

### Outcomes

**Figure 3:**
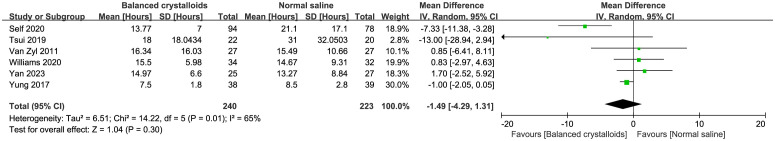
Forest plot showing the effect of balanced crystalloids versus normal saline on the time of resolution to DKA.

**Figure 4:**
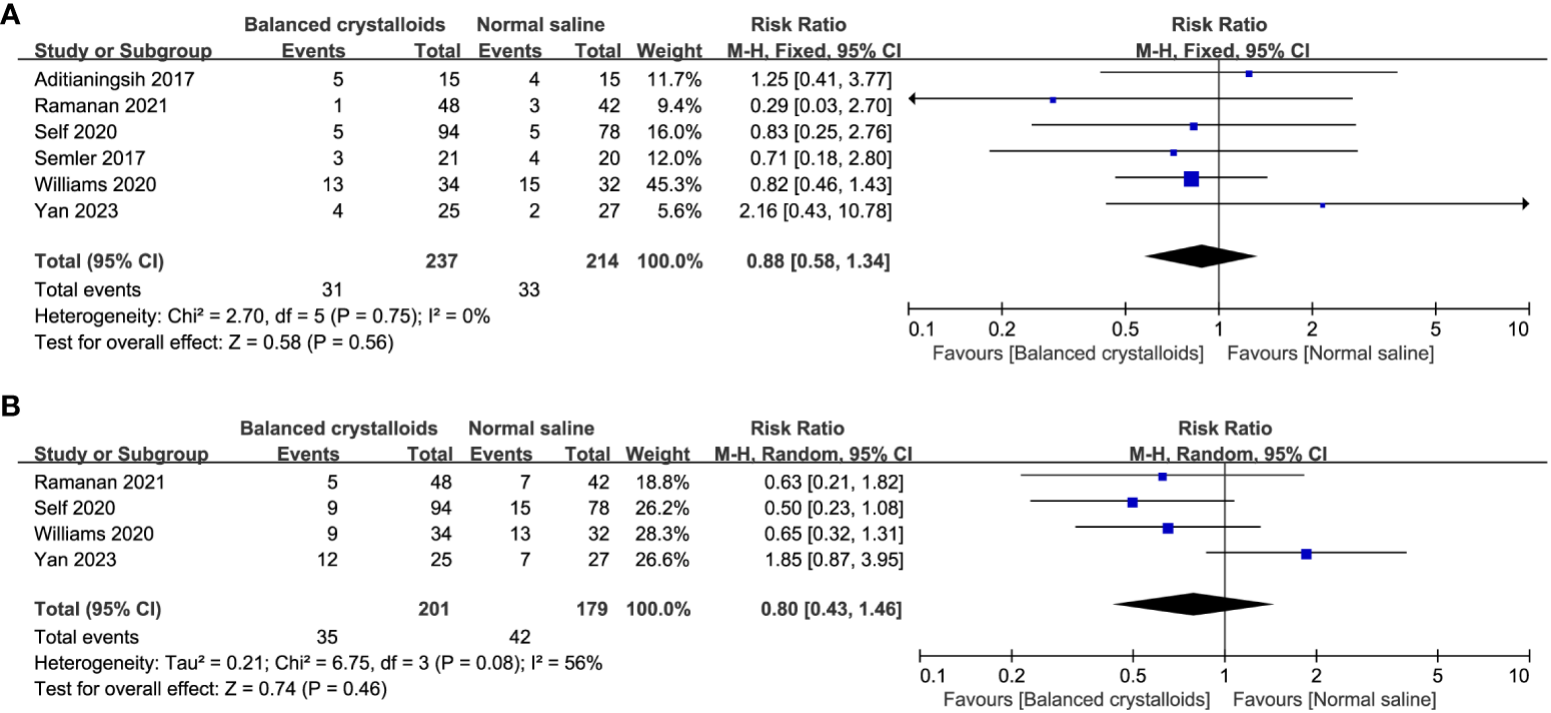
Forest plot showing the effect of balanced crystalloids versus normal saline on the **(A)** major adverse kidney events, **(B)** incidence of hypokalemia.

**Figure 5:**
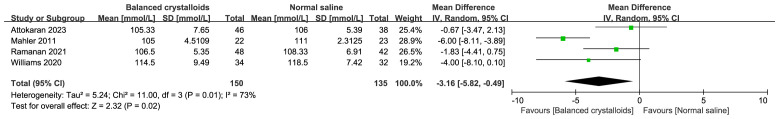
Forest plot showing the effect of balanced crystalloids versus normal saline on the post-resuscitation chloride.

Six trials reported the time to resolution of DKA and no significant difference was identified between patients receiving balanced crystalloids and normal saline (MD -1.49, 95%CI -4.29 to 1.31, P=0.30, I2 = 65%, **Figure 3**). Six trials reported the major adverse kidney events, four reported the incidence of hypokalemia, there was no significant difference between balanced crystalloids and normal saline groups for the major adverse kidney events (RR 0.88, 95%CI 0.58 to 1.34, P=0.56, I2 = 0%, **Figure 4A**) and incidence of hypokalemia (RR 0.80, 95%CI 0.43 to 1.46, P=0.46, I2 = 56%, **Figure 4B**). Four trials reported the post-resuscitation chloride and the use of balanced crystalloids could reduce the post-resuscitation chloride (MD -3.16, 95%CI -5.82 to -0.49, P=0.02, I2 = 73%, **Figure 5**).

Furthermore, we analyzed the effect of every single trial on the pooled result by omitting each study. The use of balanced crystalloids was relevant to the obvious decreasing in incidence of hypokalemia (RR 0.59, 95%CI 0.37 to 0.93, P=0.02, I2 = 0%) after omitting the study by Yan et al. (32) (**Supplementary Material 3**). Moreover, sensitivity analysis by excluding each study showed no significant difference for other outcomes, indicating the good robustness (**Supplementary Material 3**).

**Figure 6:**
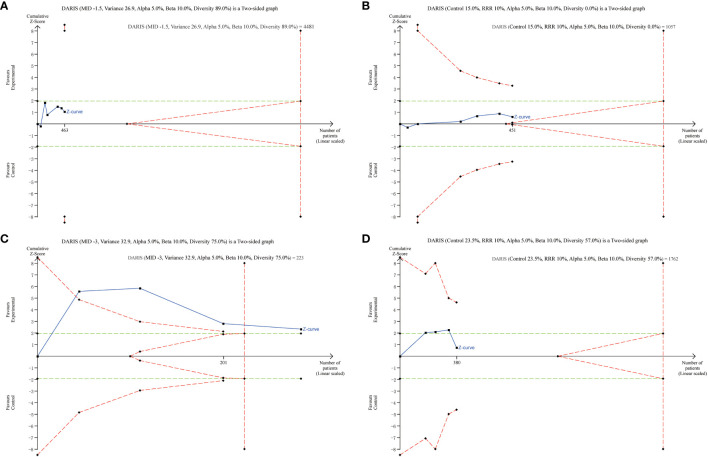
Trial Sequential Analysis of Clinical Outcomes. **(A)** time to resolution of DKA (6 studies, n=463); **(B)** major adverse kidney events (6 studies, n=451); **(C)** post-resuscitation chloride (4 studies, n=285); **(D)** incidence of hypokalemia (4 studies, n=380). The Z curve in blue measures the treatment effect (pooled relative risk). The parallel lines in green are the boundaries of conventional meta-analysis (alpha 5%), and the boundaries of benefit and harm are boundaries of conventional meta-analysis adjusted for between-trial heterogeneity and multiple statistical testing (TSA boundaries). A treatment effect outside the TSA boundaries of benefit/harm indicates reliable evidence for a treatment effect, and a treatment effect within the futility zone (the triangle between the parallel lines) indicates reliable evidence of no treatment effect.

Results of TSA are presented in **Figure 6**, showing that the current systematic review did not achieve the required information sizes to detect the pre-specified effect sizes for time to resolution of DKA, major adverse kidney events, and incidence of hypokalemia, indicating that more trials are required for a definitive conclusion for these outcomes. Furthermore, the TSA confirmed the use of balanced crystalloids was associated a significant reduction in the post-resuscitation chloride with high certainty.

## Discussion

Although there have been growing meta-analyses compares the effect of balanced crystalloids with normal saline among emergency and critical patients (9, 33–40). There is relatively limited evidence for patients with DKA. Therefore, in this meta-analysis, we comprehensively reviewed 11 RCTs to directly compare the balanced crystalloids versus normal saline in patients with DKA. Overall, compared with normal saline, the use of balanced crystalloids has no effect on the time to resolution of DKA, major adverse kidney events, and incidence of hypokalemia. However, we found that the treatment of DKA with balanced crystalloids compared to normal saline may reduce the post-resuscitation chloride. Furthermore, TSA indicated that more trials are needed to further confirm these findings.

To the best of our knowledge, this study is the most comprehensive meta-analysis of RCTs to compare the effect of balanced crystalloids with normal saline as fluid therapy in patients with DKA. Some of the findings of our meta-analysis are consist with the most recent meta-analysis by Tamzil et al. (41) that there was no significant difference in the duration of DKA resolution and acute renal failure. Conversely, Catahay et al. (42) analyzed 3 RCTs and indicated that the use of balanced crystalloids was associated with faster rates of DKA resolution compared to normal saline. However, Catahay et al. (42) only focused on the adult population and one single outcome, thus excluding other eligible trials and affecting the results.

Furthermore, our investigation revealed that the utilization of balanced crystalloids could reduce post-resuscitation chloride levels in patients with DKA. This suggests that opting for balanced crystalloids as resuscitation fluids may decrease the incidence of hyperchloremia in DKA patients. The diminished serum chloride levels observed in the balanced crystalloids group align with their lower chloride content, as these solutions emulate the plasma concentration of electrolytes. In contrast, the elevated serum chloride levels associated with normal saline could pose a risk of exacerbating hyperchloremia, thereby worsening metabolic acidosis and increasing the likelihood of acute kidney injury. Such complications may contribute to prolonged hospital stays, attributed to the deterioration of acidosis in DKA (43).

Although our meta−analysis did not reveal any noteworthy difference in the time to resolution of DKA or the major adverse kidney events between balanced crystalloids and normal saline groups, it is imperative to acknowledge potential contributing factors. These may include limitations associated with the relatively small sample size, variations in criteria for DKA resolution and adverse kidney events, or the absence of measurements related to ketone or anion gap resolution.

Our discoveries stem from an exhaustive and methodical exploration of the literature, wherein studies were rigorously identified via an exhaustive search methodology. Our inclusion criteria encompassed patients of diverse ages exhibiting varying comorbidities or etiologies of DKA at baseline. Two studies (26, 29) included the children with DKA, four studies (22, 25, 26, 30) included adult patients with moderate to severe DKA, while others included mild to severe DKA patients. Our findings should be taken with caution, even though the inclusion of all these studies allowed us to cover a wide range of patients. Criteria for the resolution of DKA varied substantially between studies: three studies (27, 28, 32) adhered to the criteria outlined in the American Diabetes Association (ADA) Consensus Statement on hyperglycemic crises 2009 criteria (44), one (23) adhered to the ADA Consensus Statement 2006 criteria (45), and another study (29) adopted criteria in accordance with the International Society of Pediatric and Adolescent Diabetes Guidelines 2014 (46). Hence, the variability in criteria employed is poised to exert an impact on the observed outcomes. Regrettably, the limited quantity of studies at our disposal precluded the execution of subgroup analyses. Such analyses would have been instrumental in elucidating the translational potential of our findings across a broader spectrum of patients. In addition, when we analyzed the effect of every single trial on the pooled results, we found that after omitting the study by Yan et al. (32), the use of balanced crystalloids was relevant to the obvious decreasing in incidence of hypokalemia. Since the patients in balanced crystalloids group had higher rate of comorbidities and co-diagnoses to DKA, the difference in the baseline disease severity between groups might led to this result.

### Strength and limitations

The strength of our work lies in the comprehensive search and analysis and the predefined analysis plan for meta-analysis, all of which increase the transparency of information. Furthermore, the use of TSA enabled us to detect the risk of type-I or type-II errors in our findings. The DARIS estimated from TSA will also inform the sample size needed for adequately powered future trials.

The current study had certain limitations as well. Primarily, it is noteworthy that all incorporated trials are inherently characterized as small-sample studies (each with fewer than 100 patients per arm), thereby introducing the potential for bias associated with the small study effect (47). There should ideally be more large-scale RCTs as the majority of included studies had limited sample sizes, which might affect the power of these analyses. Secondly, various elements contribute to the heterogeneity inherent in our analysis, encompassing divergent population characteristics, diverse etiologies of DKA, and variations in the criteria employed for DKA resolution across the studies. Additionally, a broad spectrum of major adverse kidney events exhibited considerable variability, underscoring challenges in the uniformity of definitions and the inherent difficulties in the detection and reporting of adverse events. Thirdly, some important clinical outcomes such as length of hospital stay, time-to-discontinuation of insulin, and total insulin infusion were rarely recorded and analyzed in included trials, future studies should focus on these indicators of recovery. Last but not the least, the need for additional investigation persists regarding whether various facets of fluid composition and administration, such as osmolarity, temperature, and infusion speed, exert a modifying influence on the impact of crystalloid composition on clinical outcomes (48, 49). Further clinical trials on whether there is a difference in outcome between the types of balanced crystalloids used on DKA patients would also be a good addition in future studies to show whether the difference in composition between balanced crystalloids types would exhibit superiority or non-inferiority in patient outcomes when compared.

## Conclusion

In this meta-analysis, the use of balanced crystalloids among patients with DKA has no significant different effect on the time to resolution of DKA, major adverse kidney events, and incidence of hypokalemia. However, its administration might decrease the post-resuscitation chloride. More large-scale RCTs with relatively large fluid exposure among patients with DKA are needed to guide the choice of the type of fluid resuscitation.

## Data availability statement

The original contributions presented in the study are included in the article/**Supplementary Material**. Further inquiries can be directed to the corresponding author.

## Author contributions

YL: Writing – original draft, Formal analysis, Data curation. JZ: Writing – review & editing, Software, Methodology, Formal analysis. XX: Writing – review & editing, Project administration, Methodology, Data curation. XZ: Writing – original draft, Project administration.
