# Correction: Comparison of balanced crystalloids versus normal saline in patients with diabetic ketoacidosis: a meta-analysis of randomized controlled trials

**DOI:** 10.3389/fendo.2026.1795546

**Published:** 2026-02-16

**Authors:** Yuting Liu, Jianfeng Zhang, Xiaoya Xu, Xiaoyun Zou

**Affiliations:** 1 Oncology and Chemotherapy Department, The First Affiliated Hospital of Lishui University, Lishui People’s Hospital, Lishui, China; 2 Department of Orthopedics, Yunhe People’s Hospital, Yunhe, China; 3 Department of General Surgery, The First Affiliated Hospital of Lishui University, Lishui People's Hospital, Lishui, China; 4 Department of General Practice, The First Affiliated Hospital of Lishui University, Lishui People’s Hospital, Lishui, China

**Keywords:** balanced crystalloids, normal saline, diabetic ketoacidosis, meta-analysis, resuscitation

Affiliation Oncology and Chemotherapy Department, The First Affiliated Hospital of Lishui University, Lishui People's Hospital, Lishui, China was omitted for author Yuting Liu. This affiliation has now been added for author Yuting Liu. 

Affiliation Department of General Surgery, The First Affiliated Hospital of Lishui University, Lishui People's Hospital, Lishui, China was omitted for author Xiaoya Xu. This affiliation has now been added for author Xiaoya Xu. 

Affiliation Department of General Practice, Lishui People's Hospital, Lishui, China was omitted for author Xiaoyun Zou. This affiliation has now been added for author Xiaoyun Zou.

Author Xiaoyun Zou was erroneously assigned to affiliation Department of General Practice, Lishui Central Hospital, Lishui, China. This affiliation has now been removed for author Xiaoyun Zou.

The original version of this article has been updated.

