# Supplementary material for: Comparison of balanced crystalloids versus normal saline in patients with diabetic ketoacidosis: a meta-analysis of randomized controlled trials
Source: Front Endocrinol (Lausanne). 2024 May 21;15:1367916. doi: 10.3389/fendo.2024.1367916 (PMC11148269; doi:10.3389/fendo.2024.1367916)
Supplement: Supplementary file 1 [file DataSheet_1.docx]

**Supplementary Material 2: Searching strategies, parameters for trial sequential analysis, list of excluded studies with reasons, funnel plot, and sensitivity analyses**

**Pubmed 67**

#1 Diabetic Ketoacidosis [MeSH Terms] OR Diabetic Ketoacidosis [Title/Abstract] OR Diabetic [Title/Abstract] OR Ketoacidosis [Title/Abstract]

#2 buffered [Title/Abstract] OR balanced [Title/Abstract] OR Plasmalyte [Title/Abstract] OR Ringer's Lactate [Title/Abstract] OR lactated Ringer’s [Title/Abstract] OR Ringer's Lactate [MeSH Terms]

#3 Saline Solution [MeSH Terms] OR Sodium Chloride [MeSH Terms] OR Saline Solution [Title/Abstract] OR Sodium Chloride [Title/Abstract] OR Saline [Title/Abstract] OR 0.9% NaCl [Title/Abstract]

#4 randomized controlled trial [MeSH Terms] OR random* [Title/Abstract]

#1 AND #2 AND #3 AND #4

**Embase 36**

#1 ‘Diabetic Ketoacidosis’:ti,ab,kw OR ‘Diabetic’:ti,ab,kw OR ' Diabetic Ketoacidosis '/exp OR ‘Ketoacidosis’:ti,ab,kw

#2 ' buffered ':ti,ab,kw OR ' plasmalyte '/exp OR ' ringer lactate '/exp OR ' lactate ringer solution '/exp OR ' balanced ':ti,ab,kw OR ' Plasmalyte ':ti,ab,kw OR ' Ringer Lactate ':ti,ab,kw OR ' lactated Ringer ':ti,ab,kw

#3 ' Saline Solution ':ti,ab,kw OR ' Saline Solution '/exp OR ' Normal Saline '/exp OR ' Normal Saline ':ti,ab,kw OR ' Sodium Chloride ':ti,ab,kw OR ' 0.9% NaCl ':ti,ab,kw

#4 'randomized controlled trial'/de OR 'randomized controlled trial'/exp OR ' randomized ':ti,ab,kw

#1 AND #2 AND #3 AND #4

**Scopus 76**

#1 TITLE-ABS-KEY (Diabetic Ketoacidosis) OR TITLE-ABS-KEY (Diabetic) OR TITLE-ABS-KEY (Ketoacidosis)

#2 TITLE-ABS-KEY (buffered) OR TITLE-ABS-KEY (balanced) OR TITLE-ABS-KEY (Plasmalyte) OR TITLE-ABS-KEY (Ringer's Lactate) OR TITLE-ABS-KEY (lactated Ringer’s)

#3 TITLE-ABS-KEY (Saline Solution) OR TITLE-ABS-KEY (Sodium Chloride) OR TITLE-ABS-KEY (Saline) OR TITLE-ABS-KEY (0.9% NaCl)

#4 TITLE-ABS-KEY (randomized) OR TITLE-ABS-KEY (random) OR TITLE-ABS-KEY (randomised)

#1 AND #2 AND #3 AND #4

**Cochrane Library 38**

#1 (Diabetic Ketoacidosis):ti,ab,kw OR (Diabetic):ti,ab,kw OR (Ketoacidosis):ti,ab,kw

#2 (buffered):ti,ab,kw OR (balanced):ti,ab,kw OR (Plasmalyte):ti,ab,kw OR (Ringer's Lactate):ti,ab,kw OR (lactated Ringer’s):ti,ab,kw

#3 (Saline Solution):ti,ab,kw OR (Sodium Chloride):ti,ab,kw OR (Saline):ti,ab,kw OR (icu):ti,ab,kw OR (0.9% NaCl):ti,ab,kw

#4 (randomized):ti,ab,kw OR (randomised):ti,ab,kw OR (random):ti,ab,kw

#1 AND #2 AND #3 AND #4

**Trial Sequential Analysis**

The following parameters for trial sequential analysis (TSA) were pre-specified: alpha 5%, beta 10% (power 90%), and the DerSimonian–Laird random effect model. Between-trial heterogeneity was adjusted by the diversity-estimate. A continuity correction factor of 0.5 was added in case of zero events. The effect sizes for categorical outcomes (major adverse kidney events, and incidence of hypokalemia) were 10%, and this was based on the clinically meaningful and realistic magnitude. For continuous outcomes (time to resolution of DKA, post-resuscitation chloride), the minimal important difference was based on the clinically meaningful and realistic magnitude. For categorical outcomes, the proportion of events in the control population was based on the pooled observed event rate of the updated meta-analysis. For continuous outcomes, the variance was based on the pooled observed standard deviation of the current updated meta-analysis. This approach will maximize the generalizability of the results.

**List of excluded studies with reasons**

| **Author, year and reference** | **Reason for exclusion** |
| --- | --- |
| Tamzil et al. (2023) [1] | Review, meta-analysis, or protocol |
| Yan et al. (2023) [2] | Review, meta-analysis, or protocol |
| Raman et al. (2023) [3] | Participants were not diabetic ketoacidosis patients |
| Othman et al. (2023) [4] | Review, meta-analysis, or protocol |
| Carrillo et al. (2022) [5] | Non-randomized controlled trial |
| Catahay et al. (2022) [6] | Review, meta-analysis, or protocol |
| Finfer et al. (2022) [7] | Participants were not diabetic ketoacidosis patients |
| Alghamdi et al. (2022) [8] | Review, meta-analysis, or protocol |
| Zampieri et al. (2021) [9] | Participants were not diabetic ketoacidosis patients |
| Bergmann et al. (2021) [10] | Non-randomized controlled trial |
| Friederich et al. (2019) [11] | No concerned outcomes |
| Self et al. (2018) [12] | No concerned outcomes |
| Semler et al. (2018) [13] | Participants were not diabetic ketoacidosis patients |
| Young et al. (2015) [14] | Participants were not diabetic ketoacidosis patients |
| Nath et al. (2015) [15] | Participants were not diabetic ketoacidosis patients |
| Hsia et al. (2015) [16] | Improper intervention or control methods |
| Billiodeaux et al. (2014) [17] | Non-randomized controlled trial |
| Roquilly et al. (2013) [18] | Participants were not diabetic ketoacidosis patients |
| Annane et al. (2013) [19] | Improper intervention or control methods |
| Chua et al. (2012) [20] | Non-randomized controlled trial |
| Chin et al. (2006) [21] | Participants were not diabetic ketoacidosis patients |

Reference:

1. Tamzil R, Yaacob N, Noor NM, Baharuddin KA: **Comparing the clinical effects of balanced electrolyte solutions versus normal saline in managing diabetic ketoacidosis: A systematic review and meta-analyses**. *Turk J Emerg Med* 2023, **23**(3):131-138.

2. Yan JW, Slim A, Van Aarsen K, Choi YH, Byrne C, Poonai N, Collins H, Clemens KK: **Balanced crystalloids (RInger's lactate) versus normal Saline in adults with diabetic Ketoacidosis in the Emergency Department (BRISK-ED): a protocol for a pilot randomized controlled trial**. *Pilot Feasibility Stud* 2023, **9**(1):121.

3. Raman S, Gibbons KS, Mattke A, Schibler A, Trnka P, Kennedy M, Le Marsney R, Schlapbach LJ: **Effect of Saline vs Gluconate/Acetate-Buffered Solution vs Lactate-Buffered Solution on Serum Chloride Among Children in the Pediatric Intensive Care Unit: The SPLYT-P Randomized Clinical Trial**. *JAMA Pediatr* 2023, **177**(2):122-131.

4. Othman MI, Nashwan AJ, Alfayoumi M, Khatib M, Abujaber AA: **Plasma-Lyte-148 Versus Normal Saline 0.9% in Diabetic Ketoacidosis Management: A Review**. *Cureus* 2023, **15**(6):e41079.

5. Carrillo AR, Elwood K, Werth C, Mitchell J, Sarangarm P: **Balanced Crystalloid Versus Normal Saline as Resuscitative Fluid in Diabetic Ketoacidosis**. *Ann Pharmacother* 2022, **56**(9):998-1006.

6. Catahay JA, Polintan ET, Casimiro M, Notarte KI, Velasco JV, Ver AT, Pastrana A, Macaranas I, Patarroyo-Aponte G, Lo KB: **Balanced electrolyte solutions versus isotonic saline in adult patients with diabetic ketoacidosis: A systematic review and meta-analysis**. *Heart Lung* 2022, **54**:74-79.

7. Finfer S, Micallef S, Hammond N, Navarra L, Bellomo R, Billot L, Delaney A, Gallagher M, Gattas D, Li Q *et al*: **Balanced Multielectrolyte Solution versus Saline in Critically Ill Adults**. *N Engl J Med* 2022, **386**(9):815-826.

8. Alghamdi NA, Major P, Chaudhuri D, Tsui J, Brown B, Self WH, Semler MW, Ramanan M, Rochwerg B: **Saline Compared to Balanced Crystalloid in Patients With Diabetic Ketoacidosis: A Systematic Review and Meta-Analysis of Randomized Controlled Trials**. *Crit Care Explor* 2022, **4**(1):e0613.

9. Zampieri FG, Machado FR, Biondi RS, Freitas FGR, Veiga VC, Figueiredo RC, Lovato WJ, Amêndola CP, Serpa-Neto A, Paranhos JLR *et al*: **Effect of Intravenous Fluid Treatment With a Balanced Solution vs 0.9% Saline Solution on Mortality in Critically Ill Patients: The BaSICS Randomized Clinical Trial**. *Jama* 2021, **326**(9):1-12.

10. Bergmann KR, Abuzzahab MJ, Nowak J, Arms J, Cutler G, Christensen E, Finch M, Kharbanda A: **Resuscitation With Ringer's Lactate Compared With Normal Saline for Pediatric Diabetic Ketoacidosis**. *Pediatr Emerg Care* 2021, **37**(5):e236-e242.

11. Friederich A, Martin N, Swanson MB, Faine BA, Mohr NM: **Normal Saline Solution and Lactated Ringer's Solution Have a Similar Effect on Quality of Recovery: A Randomized Controlled Trial**. *Ann Emerg Med* 2019, **73**(2):160-169.

12. Self WH, Semler MW, Wanderer JP, Wang L, Byrne DW, Collins SP, Slovis CM, Lindsell CJ, Ehrenfeld JM, Siew ED *et al*: **Balanced Crystalloids versus Saline in Noncritically Ill Adults**. *N Engl J Med* 2018, **378**(9):819-828.

13. Semler MW, Self WH, Wanderer JP, Ehrenfeld JM, Wang L, Byrne DW, Stollings JL, Kumar AB, Hughes CG, Hernandez A *et al*: **Balanced Crystalloids versus Saline in Critically Ill Adults**. *N Engl J Med* 2018, **378**(9):829-839.

14. Young P, Bailey M, Beasley R, Henderson S, Mackle D, McArthur C, McGuinness S, Mehrtens J, Myburgh J, Psirides A *et al*: **Effect of a Buffered Crystalloid Solution vs Saline on Acute Kidney Injury Among Patients in the Intensive Care Unit: The SPLIT Randomized Clinical Trial**. *Jama* 2015, **314**(16):1701-1710.

15. Nath SS, Pawar ST, Ansari F, Debashis R: **Balanced hydroxyethyl starch solution and hyperglycaemia in non diabetics - a prospective, randomized and controlled study**. *Anaesthesiol Intensive Ther* 2015, **47**(2):134-137.

16. Hsia DS, Tarai SG, Alimi A, Coss-Bu JA, Haymond MW: **Fluid management in pediatric patients with DKA and rates of suspected clinical cerebral edema**. *Pediatr Diabetes* 2015, **16**(5):338-344.

17. Billiodeaux ST, Samuelson CG, Willett O, Arulkumar S, Thomas D, Hamilton CS, Jain SK, Mosieri C, Fox CJ: **Intraoperative and Postoperative Blood Glucose Concentrations in Diabetic Surgical Patients Receiving Lactated Ringer's Versus Normal Saline: A Retrospective Review of Medical Records**. *Ochsner J* 2014, **14**(2):175-178.

18. Roquilly A, Loutrel O, Cinotti R, Rosenczweig E, Flet L, Mahe PJ, Dumont R, Marie Chupin A, Peneau C, Lejus C *et al*: **Balanced versus chloride-rich solutions for fluid resuscitation in brain-injured patients: a randomised double-blind pilot study**. *Crit Care* 2013, **17**(2):R77.

19. Annane D, Siami S, Jaber S, Martin C, Elatrous S, Declère AD, Preiser JC, Outin H, Troché G, Charpentier C *et al*: **Effects of fluid resuscitation with colloids vs crystalloids on mortality in critically ill patients presenting with hypovolemic shock: the CRISTAL randomized trial**. *Jama* 2013, **310**(17):1809-1817.

20. Chua HR, Venkatesh B, Stachowski E, Schneider AG, Perkins K, Ladanyi S, Kruger P, Bellomo R: **Plasma-Lyte 148 vs 0.9% saline for fluid resuscitation in diabetic ketoacidosis**. *J Crit Care* 2012, **27**(2):138-145.

21. Chin KJ, Macachor J, Ong KC, Ong BC: **A comparison of 5% dextrose in 0.9% normal saline versus non-dextrose-containing crystalloids as the initial intravenous replacement fluid in elective surgery**. *Anaesth Intensive Care* 2006, **34**(5):613-617.

**Detailed description of quality assessment**

| Mahler 2011 |  |
| --- | --- |
| Random sequence generation | Low (institution's research pharmacy provided blinded study fluids according to the randomization schedule) |
| Allocation concealment | Unclear (deficient information) |
| Blinding of participants and personnel | Low (researchers, clinicians, patients were unaware of the group assignments) |
| Blinding of outcome assessment | Low (outcome assessor were unaware of the group assignments) |
| Incomplete outcome data | Low |
| Selective reporting | Low |
| Other bias | Low |

| Van Zyl 2011 |  |
| --- | --- |
| Random sequence generation | Low (stratified randomization per center was done by center in blocks of 10 using a sequential numbered opaque box system) |
| Allocation concealment | Low (stratified randomization per center was done by center in blocks of 10 using a sequential numbered opaque box system) |
| Blinding of participants and personnel | Low (all clinicians, patients and investigators were  blinded for the coding of resuscitation fluid) |
| Blinding of outcome assessment | Low (all clinicians, patients and investigators were  blinded for the coding of resuscitation fluid) |
| Incomplete outcome data | Low |
| Selective reporting | Low |
| Other bias | Low |

| Semler 2017 |  |
| --- | --- |
| Random sequence generation | Low (used computer-generated simple randomization) |
| Allocation concealment | Low (cluster-randomized, cluster-crossover design) |
| Blinding of participants and personnel | High (open-label study) |
| Blinding of outcome assessment | Low (outcome assessor were unaware of the group assignments) |
| Incomplete outcome data | Low |
| Selective reporting | Low |
| Other bias | Low |

| Yung 2017 |  |
| --- | --- |
| Random sequence generation | Low (used randomly permuted blocks) |
| Allocation concealment | Low (used double, opaque envelopes to allocate treatment) |
| Blinding of participants and personnel | Low (researchers, clinicians, patients were unaware of the group assignments) |
| Blinding of outcome assessment | Low (outcome assessor were unaware of the group assignments) |
| Incomplete outcome data | Low |
| Selective reporting | Low |
| Other bias | Low |

| Aditianingsih 2017 |  |
| --- | --- |
| Random sequence generation | Unclear (deficient information) |
| Allocation concealment | Unclear (deficient information) |
| Blinding of participants and personnel | High (single-blind study) |
| Blinding of outcome assessment | Unclear (deficient information) |
| Incomplete outcome data | Low |
| Selective reporting | Low |
| Other bias | Low |

| Tsui 2019 |  |
| --- | --- |
| Random sequence generation | Unclear (deficient information) |
| Allocation concealment | Unclear (deficient information) |
| Blinding of participants and personnel | High (open-label study) |
| Blinding of outcome assessment | Unclear (deficient information) |
| Incomplete outcome data | Low |
| Selective reporting | Low |
| Other bias | Low |

| Self 2020 |  |
| --- | --- |
| Random sequence generation | Low (cluster randomized, multiple crossover design) |
| Allocation concealment | Low (cluster randomized, multiple crossover design) |
| Blinding of participants and personnel | Low (double-blind study) |
| Blinding of outcome assessment | Low (outcome assessor were unaware of the group assignments) |
| Incomplete outcome data | Low |
| Selective reporting | Low |
| Other bias | Low |

| Williams 2020 |  |
| --- | --- |
| Random sequence generation | Low (randomization scheme number was generated by a  person not involved in the study using a web-based program) |
| Allocation concealment | Low (randomization scheme number was generated by a  person not involved in the study using a web-based program) |
| Blinding of participants and personnel | Low (patients and treating physicians were blinded to the treatment) |
| Blinding of outcome assessment | Low (outcome assessor were unaware of the group assignments) |
| Incomplete outcome data | Low |
| Selective reporting | Low |
| Other bias | Low |

| Ramanan 2021 |  |
| --- | --- |
| Random sequence generation | Low (allocated using randomly generated computer tables) |
| Allocation concealment | Low (allocated using randomly generated computer tables) |
| Blinding of participants and personnel | High (open-label study) |
| Blinding of outcome assessment | Low (outcome assessor were unaware of the group assignments) |
| Incomplete outcome data | Low |
| Selective reporting | Low |
| Other bias | Low |

| Yan 2023 |  |
| --- | --- |
| Random sequence generation | Low (used computer-generated simple randomization) |
| Allocation concealment | Low (pharmacy prepared an opaque covering over each fluid bag within study kits) |
| Blinding of participants and personnel | Low (patients, clinical team, and outcome assessors were blinded to allocation group) |
| Blinding of outcome assessment | Low (patients, clinical team, and outcome assessors were blinded to allocation group) |
| Incomplete outcome data | Low |
| Selective reporting | Low |
| Other bias | Low |

| Attokaran 2023 |  |
| --- | --- |
| Random sequence generation | Low (used randomly generated computer tables) |
| Allocation concealment | Low (used randomly generated computer tables) |
| Blinding of participants and personnel | High (open-label study) |
| Blinding of outcome assessment | Low (outcome assessor were unaware of the group assignments) |
| Incomplete outcome data | Low |
| Selective reporting | Low |
| Other bias | Low |


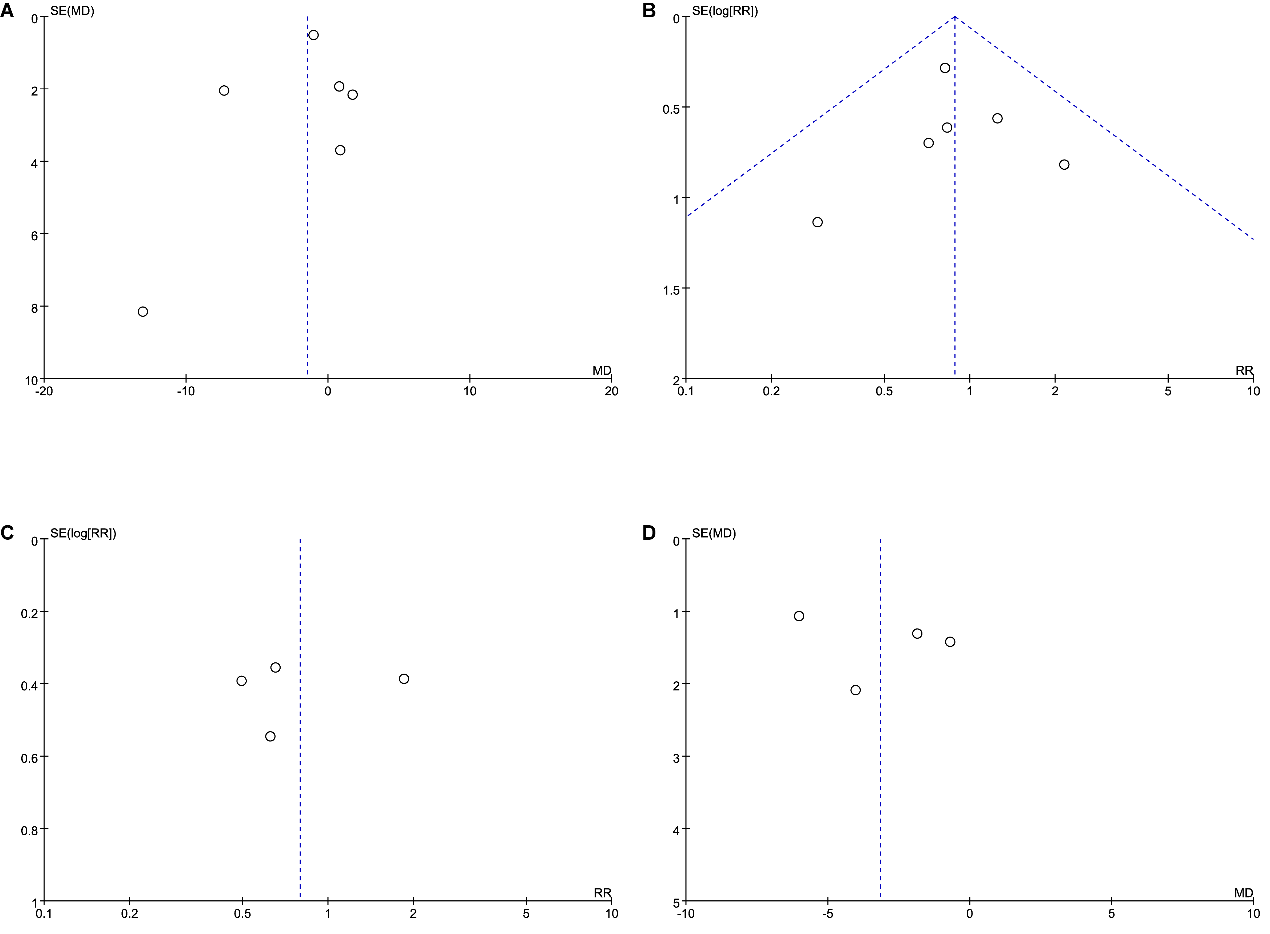


**Figure 1: Funnel plot for (A) time to resolution of DKA, (B) major adverse kidney events, (C) incidence of hypokalemia, (D) post-resuscitation chloride**


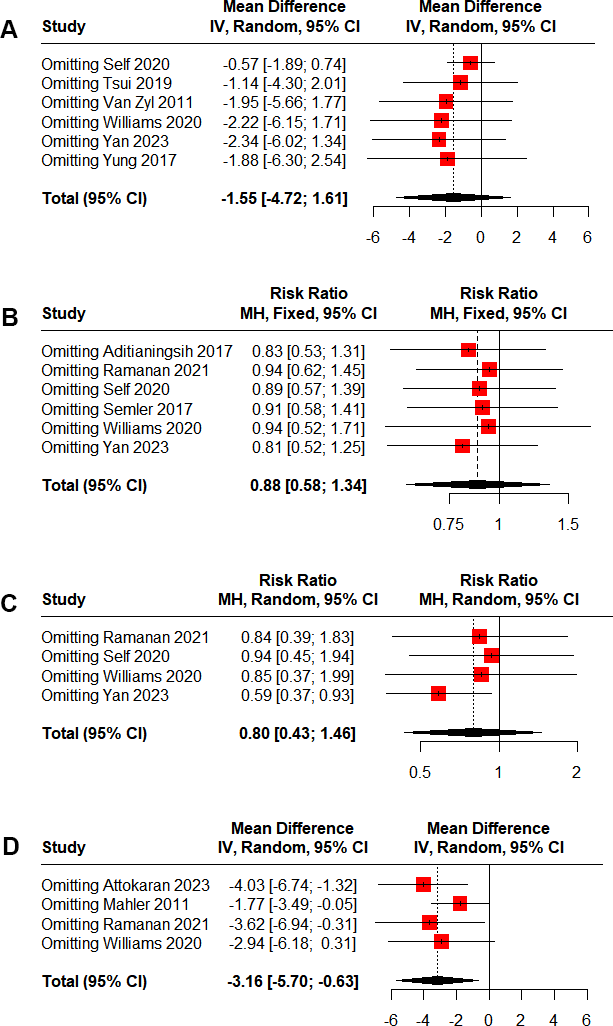


**Figure 2: Sensitivity analyses for (A) time to resolution of DKA, (B) major adverse kidney events, (C) incidence of hypokalemia, (D) post-resuscitation chloride**
